# Case Report: Mini-endoscopic combined intrarenal surgery in an en-bloc kidney transplant

**DOI:** 10.1186/s12301-021-00249-4

**Published:** 2021-10-26

**Authors:** Diego Santillán, Jordan Ceferino Scherñuk Schroh, Patricia Andrea Gutierrez, Franco Thomas, Federico Ignacio Tirapegui, Juan Moldes, Christian Cristallo, Mariano Sebastian González

**Affiliations:** Hospital Italiano de Buenos Aires, Peron, 4190, (C1181ACH), Buenos Aires, Argentina

**Keywords:** Endoscopic combined intrarenal surgery, Kidney calculi, Ureteral access sheath, Endourology

## Abstract

**Background:**

Overall incidence of stones in kidney transplant recipients is 1%. En-bloc kidney transplant is a rare anatomical condition in which kidney stones treatment can be extremely difficult to treat. As far as we know, no cases of staghorn calculi in en-bloc kidney transplant have been published so far.

**Case presentation:**

A 27-year-old woman presented to the Emergency Department because of asthenia, adynamia and weight loss associated with lower urinary tract symptoms and subfebrile temperature. Ten years before, she had undergone an en-bloc kidney transplant because of end-stage renal disease secondary to perinatal asphyxia syndrome. One kidney was implanted capo-volta in the right iliac fossa and the other one in the right flank. NCCT scan showed incomplete staghorn calculi in the iliac fossa transplanted kidney. Besides, severe dilation of the native and the right flank transplanted kidney, due to two ureteral stones of 6 and 7 mm impacted in the uretero-ureteral anastomosis, was found. After hospital admission and under ceftriaxone prophylaxis, an attempt to perform primary RIRS following our COVID protocol was carried out. Nevertheless, we ended up placing a JJ stent because once the guidewire passed through the ureteral stones, purulent material came out from the ureteral orifice. She stayed 9 days in-hospital for management of postobstructive polyuria and was discharged with oral antibiotics. Three weeks afterward, we removed the stent and performed flexible ureteroscopy and holmium laser lithotripsy of the ureteral stones. In the same procedure, we performed Mini-ECIRS (21 French) previous ultrasound-guided upper pole puncture. Postoperative NCCT scan showed neither residual fragments nor operative complications.

**Conclusion:**

This is the first clinical case reporting Mini-ECIRS in a patient with an en-bloc kidney transplant. This endourological approach seems to be a feasible, safe and effective approach to treat stones in this anatomically challenging condition.

## Background

Overall incidence of kidney stones in kidney transplant recipients is 1% [1]. Despite its rarity, allograft lithiasis can result in significant morbidity and treatment of these patients can be particularly challenging. Due to ectopic location of the kidney allograft, bones of the pelvis may preclude visualization of stones and may also attenuate the effect of extracorporeal shock wave lithotripsy (ESWL). Moreover, ectopic ureterovesical implantation implies that retrograde approaches are often difficult or not feasible. Percutaneous nephrolithotomy (PNL) is therefore widely used for renal transplant lithiasis, but perinephric scarring can increase the difficulty of puncturing and tract dilation, increasing the risk of bleeding.

The aim of this report is to present a clinical case in which Mini-ECIRS (endoscopic combined intrarenal surgery) was successfully used for the treatment of ureteral and staghorn calculi in a patient with a history of en-bloc kidney transplant. To our knowledge, this would be the first reported case on Mini-ECIRS in such a challenging condition.

## Case presentation

### Clinical history and diagnosis

A 27-year-old woman presented to the Emergency Department complaining of asthenia, adynamia and weight loss associated with lower urinary tract symptoms and subfebrile temperature. Ten years before, she had undergone a pediatric en-bloc kidney transplant because of end-stage kidney disease secondary to perinatal asphyxia syndrome. Asphyxia can lead to multi-organ dysfunction and a redistribution of cardiac output to maintain cerebral, cardiac and adrenal perfusion while potentially compromising renal, gastrointestinal and skin perfusion as circulatory response.

**Fig. 1:**
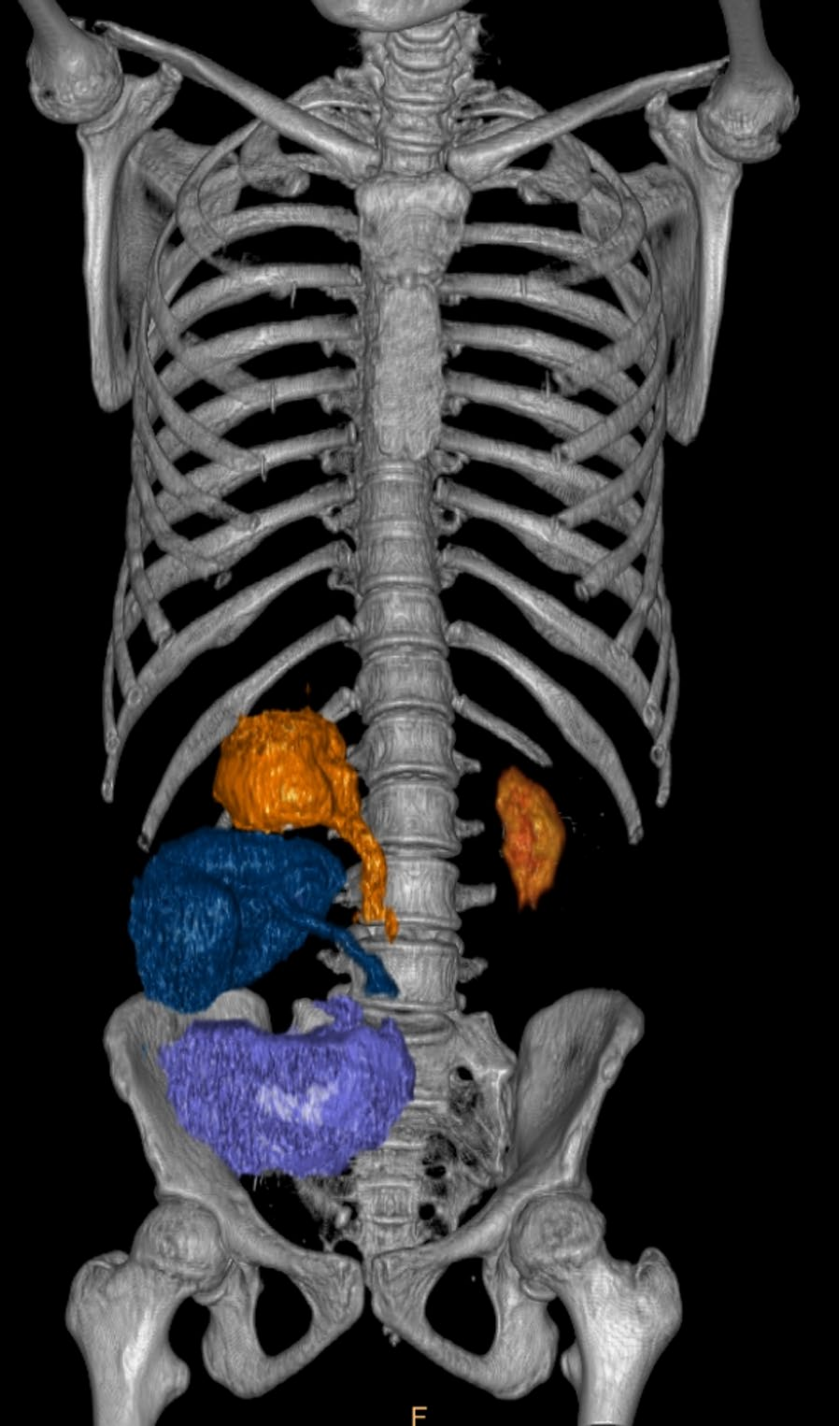
CT reconstruction showing the patient’s native kidneys (orange), the right flank transplanted kidney (blue) and the right iliac fossa transplanted kidney (violet)

One allograft was located in the right iliac fossa (upside-down implantation with ureteric anastomosis following Lich-Gregoir technique, violet in Fig. 1) and the other in the right flank (end-to-side uretero-ureteral anastomosis with native right kidney, blue and orange in Fig. 1). The en-bloc graft was made to rest on the right psoas muscle, and the graft vena cava was anastomosed terminolaterally to the recipient external iliac vein using 6–0 polypropylene suture. The graft aorta was also anastomosed terminolaterally to the right external iliac artery using 7–0 polypropylene suture.

Our patient presented other comorbidities as well such as hypertension, asymptomatic, bilateral cataracts, intellectual disability, sensorineural hearing loss and Wolff–Parkinson–White syndrome (WPW pattern or preexcitation consisting of a short PR interval and prolonged QRS with an initial slurring upstroke—“delta” wave—in the presence of sinus rhythm) with episodic palpitations and lightheadedness. Anthropometric features are : height 125 cm, weight 25 kg, BMI = 14.2. She reported 10 kg weight loss in the last 5 months due to depression symptoms associated with COVID-19 (coronavirus disease) quarantine. Her daily medication was: prednisone 4 mg, amlodipine 5 mg, atenolol 25 mg, enalapril 2.5 mg, mycophenolic acid 250 mg/250 mg and tacrolimus 2 mg/2 mg.

The main abnormalities in the laboratory data were white blood cells count 15,654/mm3, serum creatinine level 1 mg/dl (baseline: 0.6 mg/dl), lactate 3.6 mmol/l and pH 7.39.

**Fig. 2:**
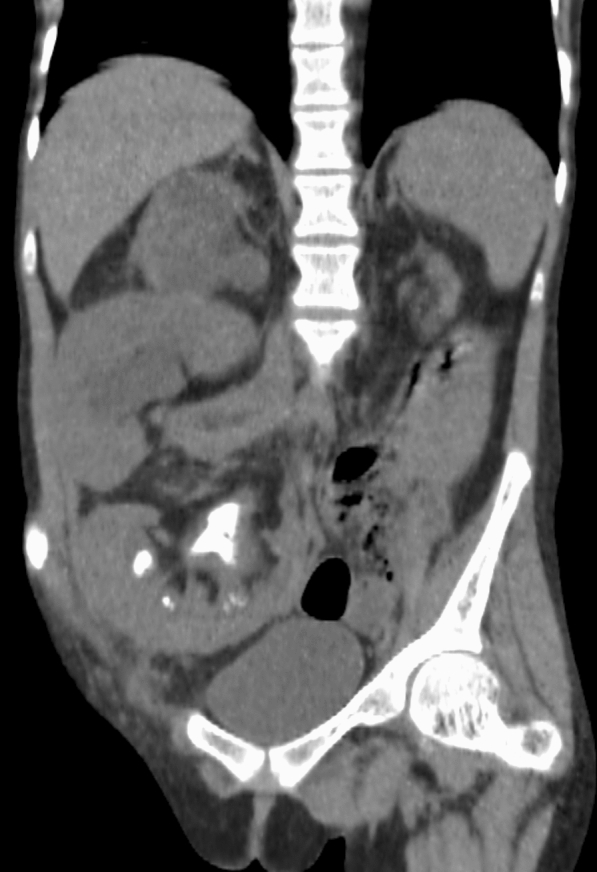
Preoperative CT scan shows incomplete staghorn calculi in right iliac fossa transplanted kidney and right native and flank transplanted kidneys dilated by ureteral stone

Non-contrast computed tomography (NCCT) scan showed staghorn calculi in the kidney allograft implanted in the right iliac fossa composed of at least 3 stones of 12.5 mm, 13.7 mm and 10 mm located in superior, medium and inferior calyx, respectively (530 Hounsfield Units) (Fig. 2). Moreover, both the right flank transplanted kidney and the right native kidney had severe pelvicalyceal dilation because of two ureteral stones of 6.7 mm and 5.9 mm impacted in the uretero-ureteral anastomosis (450 to 510 HU). Finally, a 7.2-mm stone was reported in the lower calyx of her right native kidney (457 HU).

Initial management consisted in hospital admission and empiric treatment with ceftriaxone. After 24 h of empiric ceftriaxone and following our COVID19 protocol, an attempt to perform primary URS of the ureteral stones was carried out. Nevertheless, once the guidewire (Sensor™ PTFE-Nitinol Guidewire with Hydrophilic Tip, Boston Scientific®) passed through the ureteral stones, purulent material came out from the ureteral meatus. Due to this finding, we decided to take an upper urinary tract urine sample for culture, place a double pig-tail stent and a bladder catheter, and stop the procedure. She stayed 9 days in-hospital for management of postobstructive polyuria and was discharged with oral antimicrobial agents (ciprofloxacin and fluconazole) and without the bladder catheter.

### Surgical technique and operative results

Definitive operative treatment of urolithiasis was performed three weeks thereafter. The traditional lithotomy position, in this unprecedented patient, allowed both antegrade and retrograde access. After removing ureteral stent, semirigid ureteroscopy (8Fr, Richard Wolf, Germany®) and Holmium-YAG laser lithotripsy of the ureteral stones (120 W, Lumenis®) were carried out. Retrograde intrarenal surgery (RIRS) (Flex X2, Karl Storz®) was required to treat the stone located in the inferior calyx of the native kidney. Fragmentation of the stones was executed using these laser settings: 0.8 J and 10 Hz. Fragments were removed using a nitinol stone-retrieval frontal basket (NGage, Cook Medical®).

**Fig. 3:**
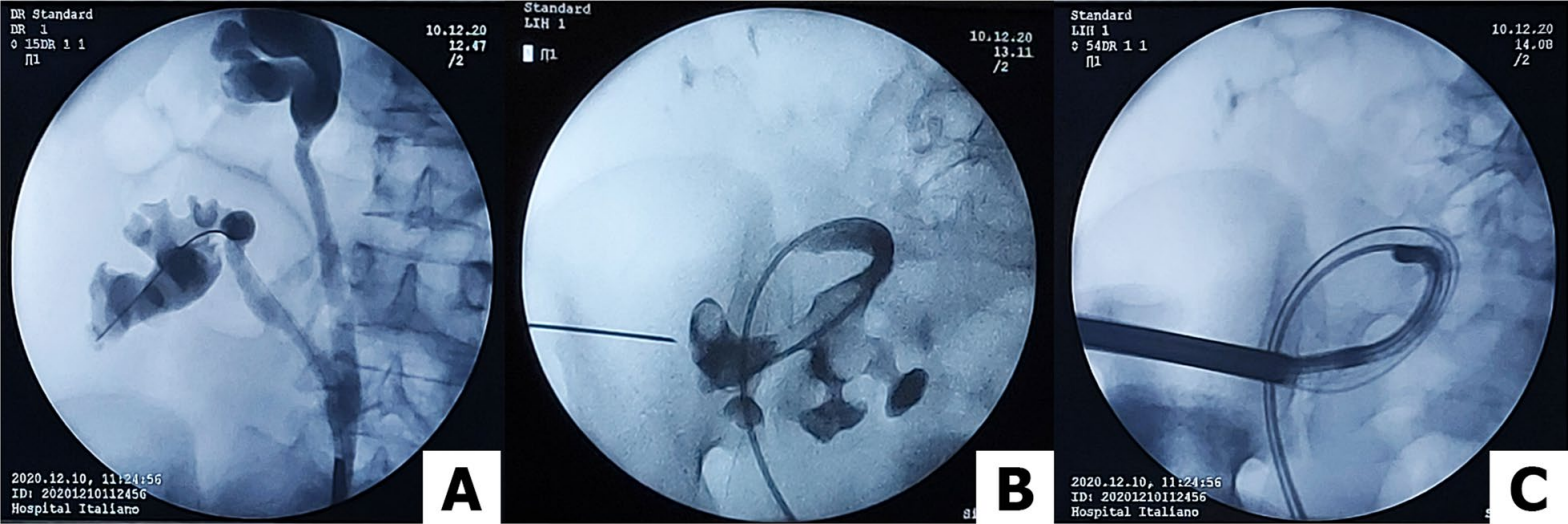
**A** Right native (upper kidney) and right flank transplanted kidneys dilated due to ureteral stones impacted in the uretero-ureteral end-to-side anastomosis. **B** Open-end ureteral catheter ascended after catheterization of the ureteroneocystostomy. Upper pole puncture of the right iliac fossa transplanted kidney under ultrasound guidance. **C** Antegrade flexible nephroscopy assessing residual fragments after finishing the percutaneous procedure

At the meantime, upper pole puncture of the right iliac fossa allograft was performed under ultrasound (US) guidance by a second endourologist. Endovision puncture was not attempted in order to protect the reusable flexible ureteroscope from an extreme proximal ureteral kinking loop (Fig. 3B).

Tract dilation was accomplished using one-shot dilator to establish a 21-Fr working channel over the guidewire. Mini-PNL (MIP Storz 12 Fr Nephroscope ®) was performed with Holmium-YAG laser dusting of the stones (settings: 0.5 J and 50 Hz). Stone fragments and dust were flushed out through the sheath by vortex effect. After most of the stone fragments had been removed, antegrade flexible nephroscopy (Flexible Fiber-Cystoscope 15Fr, Richard Wolf, Germany®) was conducted to search for residual stones in locations that were inaccessible with the rigid nephroscope (Fig. 3C). A 14-Fr 100% silicon nephrostomy tube and a bladder catheter were left in place. Operating time was 120 min. Estimated blood loss was approximately 50 cc.

**Fig. 4:**
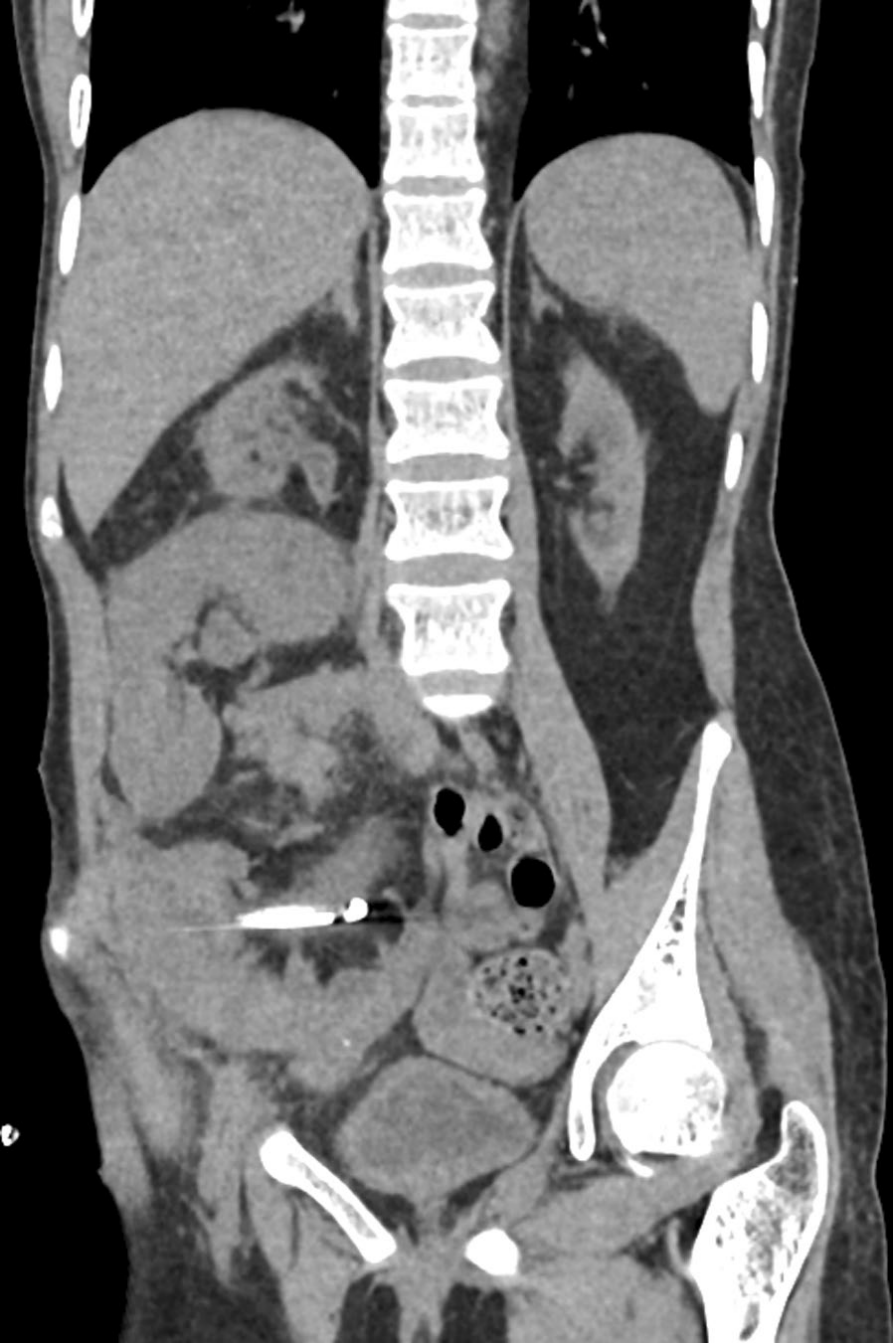
Postoperative CT scan without neither residual fragments nor subcapsular hematoma

Postoperative NCCT scan, performed 12 h postoperatively, showed neither residual fragments nor suggestive signs of immediate postoperative complications (Fig. 4). Both the nephrostomy and the bladder catheter were removed the following day. The patient remained in hospital for 3 days because of tacrolimus blood levels fluctuation. Neither early nor late onset postoperative complications occurred.

After 10 months from discharge, the patient remains stone-free. She is now under magnesium supplement and nitrofurantoin 100 mg daily prophylaxis since crystallographic analysis showed ammonium magnesium phosphate as the main component of the stones. Every six months ultrasound will be performed as follow-up.

## Literature review

En-bloc transplantation of pediatric kidneys into adult recipients (EBKT) is considered a useful strategy to increase the donor pool. Many recent studies on EBKT have documented both good short-term and long-term outcomes [2].

According to a recent meta-analysis, incidence of stones in kidney transplant recipients is 1%, much lower than in the general adult populations. This may be due to protective factors such as lower glomerular filtration rates than healthy populations or the absence of tubulointerstitial defects in kidney allografts. It can also be explained by underdiagnosis, since renal denervation makes ureteral colic a symptom unlikely to happen [1]. The latter can increase the risk of diagnostic delay until patients develop fever or acute renal failure, leading to significant morbidity and increasing the risk of allograft dysfunction.

As well as in the general population, calcium-based (calcium oxalate and calcium phosphate) stones are the most frequent type in kidney transplant recipients. Interestingly, however, struvite stones are more common in these patients [1]. Rendering stone-free is one of the main objectives when dealing with infectious stones. High oral water intake, antibiotics prophylaxis and a strict follow-up are a must to prevent new formation.

According to the latest European Association of Urology guidelines, selecting the appropriate technique for stone removal in a transplanted kidney is difficult, although management principles are similar to those applied in other single renal units. Additional factors such as transplant function, coagulative status and anatomical obstacles due to the iliacal position of the organ directly influence the surgical strategy. In a recently published article describing over 30 years of experience on the management of graft stones after renal transplantation, the most frequently used treatment modality was ESWL (43.1%), followed by active surveillance (25.4%), retrograde URS (17.6%), antegrade URS (3.9%), PCNL (3.9%), open approach with ureteral reimplantation for primary stricture (3.9%) and urine alkalization (2%) [3].

Whenever technically possible, mini-percutaneous access should be of choice in these patients. This technique provides a high stone resolution rate and a fast recovery with less parenchymal injury and limited impact on allograft function compared to standard PNL [4]. Nevertheless, when treating complex cases of renal stones with abnormal anatomy it is helpful to use combined endoscopic approaches. In fact, ECIRS standardizes the combined antegrade and retrograde approach to large and/or complex urolithiasis, using both rigid and flexible scopes [5]. In this procedure, postoperative complications are diminished by reducing the number of required percutaneous tracts, decreasing bleeding risk and lowering intrarenal pressure. Single step stone-free is also more likely compared to PNL or RIRS alone, implying less economic burden and better patients’ quality of life.

We believed the best approach for our patient was to combine a miniaturized access, to diminish the risk of complications, with retrograde and antegrade approach using flexible scopes, in order to increase stone-free rate. We performed renal puncture under US guidance so as to reduce radiation exposure. Besides, US helps to achieve a perfect transpapillary puncture when contrast cannot show the pelvicalyceal configuration such as in cases of staghorn calculi.

## Conclusion

Mini-ECIRS seems to be a feasible, safe and effective approach to treat stones in anatomically challenging conditions such as in EBKT patients.

## Data Availability

Not applicable.

## Abbreviations

BMI: Body mass index
COVID-19: Coronavirus disease
ECIRS: Endoscopic combined intrarenal surgery
ESWL: Extracorporeal shock wave lithotripsy
NCCT: Non-contrast computed tomography
PNL: Percutaneous nephrolithotomy
RIRS: Retrograde intrarenal surgery
US: Ultrasound
